# Evaluation of drug-drug interactions among patients with chronic kidney disease in a South-Eastern Nigeria tertiary hospital: a retrospective study

**DOI:** 10.11604/pamj.2017.28.199.13622

**Published:** 2017-11-03

**Authors:** Maxwell Ogochukwu Adibe, Patrick Chisom Ewelum, Kosisochi Chinwendu Amorha

**Affiliations:** 1 Department of Clinical Pharmacy and Pharmacy Management, Faculty of Pharmaceutical Sciences, University of Nigeria, Nsukka, Enugu State, Nigeria

**Keywords:** Drug-drug interactions, chronic kidney disease, Rx List drug interaction checker, UNTH

## Abstract

**Introduction:**

The risk of drug-drug interactions (DDIs) is high in patients with chronic kidney disease (CKD) necessitating dose adjustments or the avoidance of drug combinations. This study aimed to evaluate DDIs among patients with CKD in the University of Nigeria Teaching Hospital (UNTH), Enugu, South-East Nigeria.

**Methods:**

This study was a retrospective review of patients with CKD who received treatment at the nephrology unit of UNTH between January 2004 and December 2014. The drug-drug interactions (DDIs) of the prescribed drugs were classified using the RxList interaction checker. The IBM SPSS Version 21.0 was utilized for statistical analysis with P-value ≤ 0.05, considered statistically significant.

**Results:**

A total of 749 DDIs were identified from the folders of the 169 patients with CKD that were eligible. Majority were above 50 years old and in stage 4 or 5 CKD. Furosemide, lisinopril and amlodipine were the most frequently prescribed drugs and had the greatest likelihood for nephrotoxicity. The number of medications and hypertension (as co-morbidity) were significant and independent predictors of DDIs among the patients. About 70% of the drug combinations required monitoring as they fell within the “significant category” of the RxList interaction checker. The most common interactions were between lisinopril and furosemide; furosemide and calcium carbonate; lisinopril and calcium carbonate.

**Conclusion:**

The prevalence of DDIs was high among the CKD patients. Prescribers and pharmacists in Nigerian hospitals may need to pay close attention to prescriptions of patients with CKD to identify, prevent and resolve undesirable DDIs.

## Introduction

Before prescriptions are made, renal function should be considered [1]. The kidneys are responsible for the excretion of about half of all drugs and their metabolites and more than a fifth of all adverse effects of medications have either a renal cause or renal effect [2]. Notwithstanding, pharmacists and other health professionals need to be aware that not all drugs depend on the renal function. In renal impairment there may be drug toxicity due to the accumulation of medicines, especially if the medicine has a narrow therapeutic index [1]. Patients on nephrotoxic drugs need to monitor their renal function [1]. Renally-impaired patients may be candidates for drug therapy problems. A drug therapy problem (DTP) is an event or circumstance that involves drug therapy, actual or potential, and interferes with desired health outcomes [3]. DTPs could prevent or delay patients from achieving desired therapeutic goals. [4] An actual DTP is an event that has already occurred in a patient, whereas a potential DTP is an event that is likely to develop if pharmacists do not make any appropriate interventions. Adverse reactions, drug interactions and therapeutic failure are common drug-related problems [5]. Chronic Kidney Disease (CKD) refers to kidney damage with manifestations of abnormal excretion of albumin or reduced kidney function, quantified by estimated glomerular filtration rate (eGFR) persisting for greater than three months [6, 7]. Individuals with CKD are at high risk for drug interactions as they often require different classes of drugs [8, 9]. Drug interactions may increase morbidity, lead to hospitalizations and deaths [10]. A useful drug interaction tool is the Rx List drug interaction checker. It checks interacting drug ingredients, their effects and clinical significance. The drug-drug interactions (DDIs) are classified as serious, significant or minor. Serious DDIs require an alternative drug; significant DDIs require close monitoring while minor DDIs are non-significant drug-drug interactions [11]. It is hoped that this study will raise awareness on the relevant DDIs that occur among CKD patients providing health professionals with a better understanding of the topic. The knowledge would enable such undesirable interactions to be avoided. The general objective of the study was to evaluate drug-drug interactions in chronic kidney disease patients in the University of Nigeria Teaching Hospital, Enugu, South-East Nigeria (UNTH).

## Methods

**Study design and sample Population:** this study was a ten-year retrospective review of patients with chronic kidney disease who received treatment at the nephrology unit of University of Nigeria Teaching Hospital (UNTH), Ituku-Ozalla, Enugu State between January 2004 and December 2014. UNTH is located about 20 kilometers from Enugu Capital City along Enugu-Port Harcourt Express Way. This tertiary institution is a federal referral hospital that has numerous specialty units that serve patients mainly from the Eastern part of Nigeria. The study participants were patients with chronic kidney disease who fulfilled the eligibility criteria. The inclusion criteria were patients who had chronic kidney disease with known serum creatinine levels. The exclusion criteria comprised patients with glomerular filtration rate greater than 90ml/min/1.73m^2^ and those who were pregnant. A total of 169 folders of eligible patients with chronic kidney disease were included and utilized in this study.

### Data Collection

**Serum potassium level and nephrotoxicity:** Data on serum potassium level and nephrotoxicity of the prescribed drugs were based on the University of Michigan Health System (UMHS) Chronic Kidney Disease Guidelines, March 2014. [12] This guideline has lists of drugs that were known to be nephrotoxic and have potentials to decrease or increase serum potassium levels. A drug was classified and coded as being nephrotoxic or affecting serum potassium if it is listed in the guideline.

**Drug-drug interactions:** Drug-drug interactions were assessed using the RxList drug interaction platform [11]. The RxList identifies the number of interactions in a prescription and rates them into the following categories: a) Contra-indicated - Never use this combination of drugs because of high risk for dangerous interaction; b) Serious - Potential for serious interaction; regular monitoring by your doctor required or alternative medication may be needed; c) Significant - Potential for significant interaction (monitoring by your doctor is likely required); d) Minor - Interaction is unlikely, minor, or non-significant. The prescribed drugs in a prescription were entered into the RxList platform to identify drug-drug interaction pairs and their classifications. If there was an interaction, the pair of the interacting drugs was noted and coded as 1 and classified as ‘contra-indicated’, ‘serious’, ‘significant’, and ‘minor’. Documentation also included data on patient characteristics (e.g. age, gender); clinical characteristics such as the presence of co-morbidities; chronic kidney disease stage; concurrent medications.

**Data analysis:** The data were collated and analyzed using the IBM Statistical Products and Service Solutions (SPSS) for Windows, Version 21.0 (IBM Corp, Version 21.0, and Armonk, NY, USA). Descriptive statistics were used to summarize data. Linear regression was used to determine the independent predictors of drug-drug interactions. P-value ≤0.05 was considered statistically significant.

**Ethical consideration:** Ethical clearance was obtained from the Health Research and Ethics Board of UNTH prior to the commencement of this study (NHREC/05/01/2008B-FWA00002458-IRB00002323). The study was conducted based on the approved protocol.

## Results

Over half of the patients were female (52.1%) with about 54.4% above 50 years old. More than half of the patients (56.2%) were in stage 4 or 5 of chronic kidney disease. Out of 299 drugs (single and fixed combinations - a drug in different multiple fixed combinations with other drugs was coded differently and independently) prescribed, 749 drug-drug interactions were identified in the prescriptions of 169 patients' folders that were assessed. The result indicated that DDIs were about 2.5 times higher than the total independent drug prescribed. There was no ‘contra-indication’. About 70% of the drug combinations required monitoring as they fell within the “significant category” of the RxList Classification, Table 1. The prescribed drugs that made up 90% of all the drugs used in patients included furosemide, lisinopril and amlodipine, Table 2. The number of medications and presence of hypertension significantly and independently predicted drug-drug interaction, Table 3. The most common interactions were between lisinopril and furosemide; furosemide and calcium carbonate; lisinopril and calcium carbonate,Table 4. Lisinopril and furosemide were the most frequently prescribed drugs that could increase and decrease serum potassium levels respectively, Table 5 while furosemide, lisinopril and amlodipine were the most frequently prescribed nephrotoxic drugs, Table 6.

**Table 1:** Patient characteristics

Characteristics	n (%)
**Age**	
≤ 18	2 (1.2)
19 – 35	25 (14.8)
36 – 50	50 (29.6)
51 – 65	68 (40.2)
>65	24 (14.2)
**Mean age ± SD (years)**	51.03 ± 14.89
**Gender**	
Male	81 (47.9)
Female	88 (52.1)
**Drug Information**	
Total number of medications	1039
Mean number of medications ± SD	6.15 ± 1.97
**Co-morbidities**	
None	29 (17.2)
Hypertension only	39 (23.1)
Diabetes	9 (5.3)
Anaemia	4 (2.4)
Hypertension-Diabetes	37 (21.9)
Hypertension-Anaemia	6 (3.6)
Others *	45 (26.6)
**Chronic kidney disease stage**	
1	6 (3.6)
2	29 (17.2)
3a	20 (11.8)
3b	19 (11.2)
4	47 (27.8)
5	48 (28.4)
**Drug-Drug Interactions Based on the RxList Classification**	
Contra-indicated	0 (0.00)
Serious	29 (3.87)
Significant	525 (70.09)
Minor	195 (26.03)

* Others: in combination with hypertension or stand-alone (heart diseases, hyperlipidaemia, stroke, congestive heart failure, arthritis, HIV, Sepsis; urinary tract infection, dermatitis, haematuria, congenital heart diseases and benign prostrate hyperplasia)

**Table 2:** Drugs that contributed to 90% of the total drug utilized (DU_90%_)

DRUGS	n (%)
Furosemide	118 (11.48)
Lisinopril	91 (8.85)
Amlodipine	75 (7.30)
Ranitidine	70 (6.81)
Hydrochlorothiazide	68 (6.61)
Calcium Carbonate	66 (6.42)
Methyldopa	50 (4.86)
Low dose aspirin	49 (4.77)
Metoclopramide	37 (3.60)
Metolazone	26 (3.60)
Losartan	20 (1.95)
Valsartan	18 (1.75)
Simvastatin	17 (1.65)
Ferrous Sulfate	16 (1.56)
Metformin	14 (1.36)
Pioglitazone	12 (1.17)
Humulin	12 (1.17)
Omeprazole	11 (1.07)
Ferrous Fumarate	11 (1.07)
Levofloxacin	11 (1.07)
Hydralazine	10 (0.97)
Atorvastatin	10 (0.97)
Epoetin alfa	10 (0.97)
Gliclazide	9 (0.88)
Spironolactone	8 (0.78)
Tramadol	8 (0.78)
Glimepiride	8 (0.78)
Vitamin B Complex	8 (0.78)
Ferrous Gluconate	8 (0.78)
Ciprofloxacin	8 (0.78)
Metronidazole	8 (0.78)
Vitamin C	7 (0.68)
Acetaminophen	6 (0.58)
Nifedipine	6 (0.58)
Rabeprazole	6 (0.58)
Dipyridamole	6 (0.58)

**Table 3:** Predictors of drug-drug interaction

Independent Variables	ß - Coefficients	SE	t	R	R^2^	Adj R^2^	p-value
**Model 1**				0.59	0.34	0.34	
Constant	-2.036	0.696	-2.926				0.004
Number of medications	1.039	0.106	9.827				< 0.001
**Model 2**				0.61	0.37	0.36	
Constant	-2.849	0.766	-3.716				< 0.001
Number of medications	1.049	0.104	10.065				< 0.001
Presence of hypertension	1.065	0.450	2.367				0.019

Dependent variable: drug-drug interaction, Significant at p ≤ 0.05, t = t - statistic (the coefficient divided by its standard error), R = Multiple correlation coefficient, SE = Standard Error

**Table 4:** DU_70%_ Interacting Drugs

Interacting drugs	n (%)
Lisinopril_Furosemide	71 (9.06)
Furosemide_Calcium carbonate	52 (7.22)
Calcium carbonate_Lisinopril	44 (6.11)
Aspirin_Furosemide	33 (4.58)
Furosemide_Hydrochlorothiazide	32 (4.44)
Calcium carbonate_Amlodipine	31 (4.31)
Aspirin_Lisinopril	30 (4.17)
Aspirin_HCTZ	21 (2.92)
Furosemide_Metolazone	20 (2.78)
Hydrochlorothiazide_Ranitidine	18 (2.50)
Aspirin_Calcum carbonate	17 (2.36)
Hydrochlorothiazide_Calcium carbonate	16 (2.22)
Ferrous sulphate_Ranitidine	11 (1.53)
Furosemide_Valsartan	11 (1.53)
Furosemide_Metformin	10 (1.39)
Furosemide_Losartan	9 (1.25)
Hydrochlorothiazide_Losartan	9 (1.25)
Hydrochlorothiazide_Metformin	9 (1.25)
Hydrochlorothiazide_Valsartan	9 (1.25)
Metolazone_Calcium carbonate	9 (1.25)
Aspirin_Valsartan	8 (1.11)
Ferrous Sulphate_Methyldopa	8 (1.11)
Furosemide_Spironolactone	7 (0.97)
Methyldopa_Metoclopramide	7 (0.97)
Hydrochlorothiazide_Metolazone	6 (0.83)
Digoxin_Furosemide	5 (0.69)
Glimepiride_Lisinopril	5 (0.69)

**Table 5:** Drugs that affect serum potassium level

Drugs that affect serum potassium level	n (%)
**Drugs that Increase Serum Potassium Level**	
Lisinopril	88 (62.00)
Losartan	18 (12.70)
Valsartan	14 (9.90)
Spironolactone	6 (4.20)
Propranolol	6 (4.20)
Ramipril	3 (2.10)
Enalapril	2 (1.40)
Telmisartan	2 (1.40)
Irbesartan	1 (0.70)
Candesartan	1 (0.70)
Atenolol	1 (0.70)
**Drugs that Decrease Serum Potassium Level**	
Furosemide	114 (57.30)
Hydrochlorothiazide	66 (33.20)
Metolazone	15 (7.50)
Torsemide	2 (1.00)
Prednisolone	1 (0.50)
Aspirin	1 (0.50)

**Table 6:** Nephrotoxic drugs

Nephrotoxic drugs	n (%)
Furosemide	114 (26.03)
Lisinopril	89 (20.32)
Amlodipine	71 (16.21)
Aspirin	45 (10.27)
Losartan	20 (4.57)
Valsartan	18 (4.11)
Levofloxacin	12 (2.74)
Omeprazole	10 (2.28)
Spironolactone	7 (1.60)
Rabeprazole	7 (1.60)
Digoxin	6 (1.37)
Ciprofloxacin	6 (1.37)
Nifedipine	5 (1.14)
Ramipril	4 (0.91)
Torsemide	4 (0.91)
Ofloxacin	4 (0.91)
Hydralazine	4 (0.91)
Cefpodoxime	4 (0.91)
Enalapril	3 (0.68)
Cefixime	1 (0.23)
Carvedilol	1 (0.23)
Telmisartan	1 (0.23)
Candesartan	1 (0.23)
Irbesartan	1 (0.23)

## Discussion

More than half of the patients with chronic kidney disease were female, above 50 years old and in stage 4 or 5 of chronic kidney disease. Furosemide, lisinopril and amlodipine were the most utilized drugs. The number of medications and presence of hypertension significantly and independently predicted drug-drug interaction. Majority of the drug-drug interactions were significant. The most common interactions were between lisinopril and furosemide; furosemide and calcium carbonate; lisinopril and calcium carbonate. Lisinopril and furosemide were the most frequently prescribed drugs that could increase and decrease serum potassium levels, respectively. Furosemide, lisinopril and amlodipine were the most frequently prescribed drugs that were nephrotoxic. In this study, the mean age of patients with chronic kidney disease was approximately 51 years supporting earlier research works that pointed out older age as a risk factor for the occurrence of polypharmacy and the development of chronic kidney disease [1, 13]. In an Indian study among CKD patients, most of the recorded DDIs occurred in patients between 51-70 years [13]. After the age of 40 years, the glomerular filtration rate declines by about 10 mL/min every 10 years [1]. The highest occurring co-morbidity was hypertension and patients with hypertension as a co-morbid state had higher risk of drug-drug interactions. Anti-hypertensives have been indicated with a high number of DDIs [10, 13]. In a study among renal failure patients of the nephrology ward in a South Indian tertiary care hospital, calcium channel blockers and beta blockers constituted the major class of drugs involved in interactions [9]. Also, majority of the patients with stage G4 and G5 had the highest prevalence of the disease, probably because the early stage of CKD is usually asymptomatic thereby making the patients to report to the hospital when the disease has deteriorated to the later stages with symptoms. In the later stages of the disease, patients with renal insufficiency are at high risk of DDIs [13].

For every additional medication and the presence of hypertension as co-morbidity, there would be increased likelihood of DDI to occur and for the presence of hypertension as co-morbidity with/without any other co-morbidity there would be increased likelihood of DDI to occur. In a study conducted in Brazil, the probability of one drug interaction increased by 2.5 (95% CI = 2.18 to 3.03) times for each additional drug and risk factors strongly associated with drug interactions were obesity, hypertension, diabetes and advanced stage of CKD [8]. The results of multiple linear regression of another study showed a significant positive association between number of potential DDIs and the total number of medications [14]. The most frequently prescribed drugs were furosemide, lisinopril and amlodipine while the most common potential interaction was lisinopril/furosemide followed by furosemide/calcium carbonate and lisinopril/calcium carbonate. This differed from a Palestinian study where calcium carbonate was most prescribed while calcium carbonate/amlodipine and calcium carbonate/aspirin had the most common potential interaction [14]. Lisinopril could increase serum potassium level while furosemide decreases serum potassium level. These two drugs also have the risk for nephrotoxicity. Renal function monitoring is recommended for patients using medicines that are nephrotoxic [1]. In addition, furosemide is a drug with a high likelihood of causing DDIs [13].

Not all drug-drug interactions are undesirable as some can be beneficial [15]. The DDIs do not always suggest exclusion of the implicated drugs from the prescriptions of renal patients [15]. Rather, it could stress the need for appropriate dose adjustments and close monitoring to avoid possible complications such as acute hypotension and renal insufficiency. It should be noted that a potential DDI does not necessarily mean that the patient would have an actual DDI and a clinically significant effect [16]. In some cases, the patient only needs extra monitoring. Based on the Rx List interaction checker, metoclopramide/methyldopa was the only ‘serious’ interaction identified, requiring immediate discontinuation. More than half of the DDIs were ‘significant’, requiring close monitoring. This was similar to the results of another study where about 56.7% of the DDIs were significant [13]. Clinical pharmacists, as integral members of the health care team, have vital roles to play in the early detection of DDIs. This involves monitoring of patient's medication chart and active participation in clinical ward rounds [13]. The practice of rational prescribing reduces polypharmacy which is one of the major causes of DDIs [4]. Also, physicians and pharmacists should be more aware of these potential interactions and better counsel patients to ensure the informed use of drugs [14]. The monitoring of potential DDIs may promote both rational prescribing and dispensing [16]. Electronic drug interaction tools should be available in the pharmacy section of hospitals, besides reference books, journals, textbooks and the availability of the internet. Further research works should be conducted to minimize the prevalence of undesirable drug-drug interactions, especially those that could lead to nephrotoxicity. This study had a few limitations that deserve consideration while interpreting the results. It should be noted that this study may not be a true representation of the CKD population in Nigeria, especially as the study was conducted in a single tertiary healthcare facility. The DDIs presented here were mainly potential not actual drug-drug interactions. It should be noted that a potential DDI does not necessarily mean that the patient would have an actual DDI and a clinically significant effect [16]. The patient may only require extra monitoring.

## Conclusion

The prevalence of DDIs was high among the CKD patients. Lisinopril and furosemide were the most frequently prescribed drugs that were nephrotoxic, affect serum potassium levels and implicated for DDIs. Prescribers and pharmacists in Nigerian hospitals may need to pay close attention to prescriptions of patients with chronic kidney disease to identify, prevent and resolve any undesirable DDIs and other drug-related problems associated with the patients' medications.

### What is known about this topic

- The risk of drug-drug interactions is high in chronic kidney disease;
- Chronic Kidney Disease (CKD) patients may need dose adjustments or the avoidance of some drug combinations.

### What this study adds

- The prevalence of significant drug-drug interactions (DDIs) is high among renal patients. The major determinants of the DDIs are the number of medications and the presence of hypertension as co-morbidity;
- Furosemide, lisinopril and amlodipine were the most frequently prescribed drugs and had the greatest likelihood for nephrotoxicity. The most common interactions were between lisinopril and furosemide; furosemide and calcium carbonate; lisinopril and calcium carbonate.

## Competing interests

The authors declare no competing interest.
